# Blood glucose profile as a rapid method for observing Koi carp (*Cyprinus carpio*) health status - case study of ectoparasites in Blitar, Indonesia

**DOI:** 10.1590/S1984-29612023019

**Published:** 2023-04-07

**Authors:** Afiyah, Maulifa Dhea Fira, Muhammad Browijoyo Santanumurti, Mamdoh Taha Jamal, Arif Muttaqin, Sri Subekti, Putri Desi Wulan Sari

**Affiliations:** 1 Aquaculture Study Program, Faculty of Fisheries and Marine, Universitas Airlangga, Surabaya, Indonesia; 2 Department of Aquaculture, Faculty of Fisheries and Marine, Universitas Airlangga, Surabaya, Indonesia; 3 Department of Marine Biology, Faculty of Marine Sciences, King Abdulaziz University, Jeddah, Kingdom of Saudi Arabia; 4 Department of Animal Husbandry and Fisheries, Blitar, East Java, Indonesia; 5 Department of Marine, Faculty of Fisheries and Marine, Universitas Airlangga, Surabaya, Indonesia

**Keywords:** Blood identification method, fish disease, glucose, Koi carp, Método de identificação de sangue, doença de peixe, glicose, carpa Koi

## Abstract

Assessment of fish health is one of the efforts of farmers in minimizing losses due to disease. Rapid tests on fish health can be done through blood observations. This study aimed to determine the blood glucose profile of koi carp due to ectoparasite infestation from the level of blood glucose. The results showed that reported parasites from Blitar’s koi carp were *Trichodina, Dactylogyrus, Gyrodactylus, Myxobolus, Thelohanellus, Ichthyophthirius*, and *Argulus*. *Trichodina* showed the highest prevalence (100%) in this case while *Thelohanellus* was the highest intensity level (93.8±16.3). The results of blood glucose level measurement based on parasite infestation levels showed no significant difference (*p*>0.05) though the health problems caused by parasites in light, medium or heavy infestation. This research also indicated that the blood glucose profile could be used as a rapid method to detect fish health caused by parasites. We suggest that other variables such as nutritional status, life stage or feeding must be conducted to ensure the glucose role in parasite identification as a rapid method for the future work.

## Resumo

A avaliação da saúde dos peixes é um dos esforços dos produtores para minimizar as perdas por doenças. Testes rápidos de saúde de peixes podem ser feitos por meio de observações de sangue. Este estudo teve como objetivo determinar o perfil glicêmico de peixes “koi”, devido à infestação de ectoparasitos a partir do nível de glicemia. Os resultados mostraram que os parasitas relatados de peixes “koi” de Blitar foram *Trichodina*, *Dactylogyrus*, *Gyrodactylus*, *Myxobolus*, *Thelohanellus*, *Ichthyophthirius* e *Argulus*. *Trichodina* apresentou a maior prevalência (100%) neste caso, enquanto *Thelohanellus* foi o maior em nível de intensidade (93,8±16,3). Os resultados da medição do nível de glicose no sangue, com base nos níveis de infestação parasitária, não mostraram diferença significativa (p>0,05), apesar dos problemas de saúde causados ​​por parasitas em infestação leve, média ou pesada. Esta pesquisa também indicou que o perfil de glicose no sangue pode ser usado como um método rápido, para detectar a saúde dos peixes causada por parasitas. Este estudo também sugere que outras variáveis, como estado nutricional, estágio de vida ou alimentação, devem ser conduzidas para garantir o papel da glicose na identificação do parasita como um método rápido para trabalhos futuros.

## Introduction

Koi carp (*Cyprinus carpio*) is one of the commercial aquaculture species in Indonesia. Data from The Ministry of Maritime Affairs and Fisheries in 2020 showed that the export value of koi carp reached 2,775 kg with nominal 34,881 USD. Study Domasevich et al. (2022) has already stated that koi is a favorite fish among aquarists for all qualitative attributes. Therefore, many koi carp cultivation businesses are carried out in Indonesia, especially Blitar. Blitar is one of the cities with the largest koi carp production in Indonesia. Previous data stated that Blitar produces 40 million koi carp per year and the government has designed Blitar as a minapolitan area for ornamental koi carp (Abidin et al., 2019).

Unfortunately, koi carp farming activities in Blitar still have problems. In June 2020, the koi production area in Blitar was attacked by a fish disease. Many studies reported that extreme parasites attacked the koi carp in Blitar (Maftuch et al., 2018; Soelistyoadi et al., 2020; Yanuhar et al., 2021). Furthermore, the parasite case in Blitar caused mortality reaching 90% (Yanuhar et al., 2021). The infected fish became weak, experienced less motion, pale color, decreased appetite, and mortality occurred. The parasites that caused the case in Blitar recently were *Myxobolus, Argulus, Trichodina, Dactylogyrus, Gyrodactylus,* and *Ichthyophthirius multifiliis* (Kismiyati et al., 2015; Azmi et al., 2013; Mahasri et al., 2011).

One way to prevent this case is to understand the condition of the fish's blood. When fish are infected by parasites, the body's defense mechanism becomes weak so that fish become stressed. Stress levels can be determined by increasing blood glucose levels in fish (Jiang et al., 2017). Stress causes an increase in glucocorticoids which results in an increase in blood glucose levels to cope with high energy requirements (Suarez-Bregua et al., 2018). If the fish's blood glucose condition is not normal, the fish's life will be disrupted and can even cause death. Measurement of blood glucose levels can be used to diagnose stressed fish simply, effectively, and rapidly for a variety of stressors (Sulmartiwi et al., 2013; Makaras et al., 2020). Based on the description above, we conducted a study in Blitar to determine the type and number of parasites and also blood glucose levels in koi carp. This study aimed to re-introduce the method of blood glucose profile determination of koi carp due to ectoparasite infestation from the level of blood glucose.

## Materials and Methods

### Sample collection

**Figure 1:**
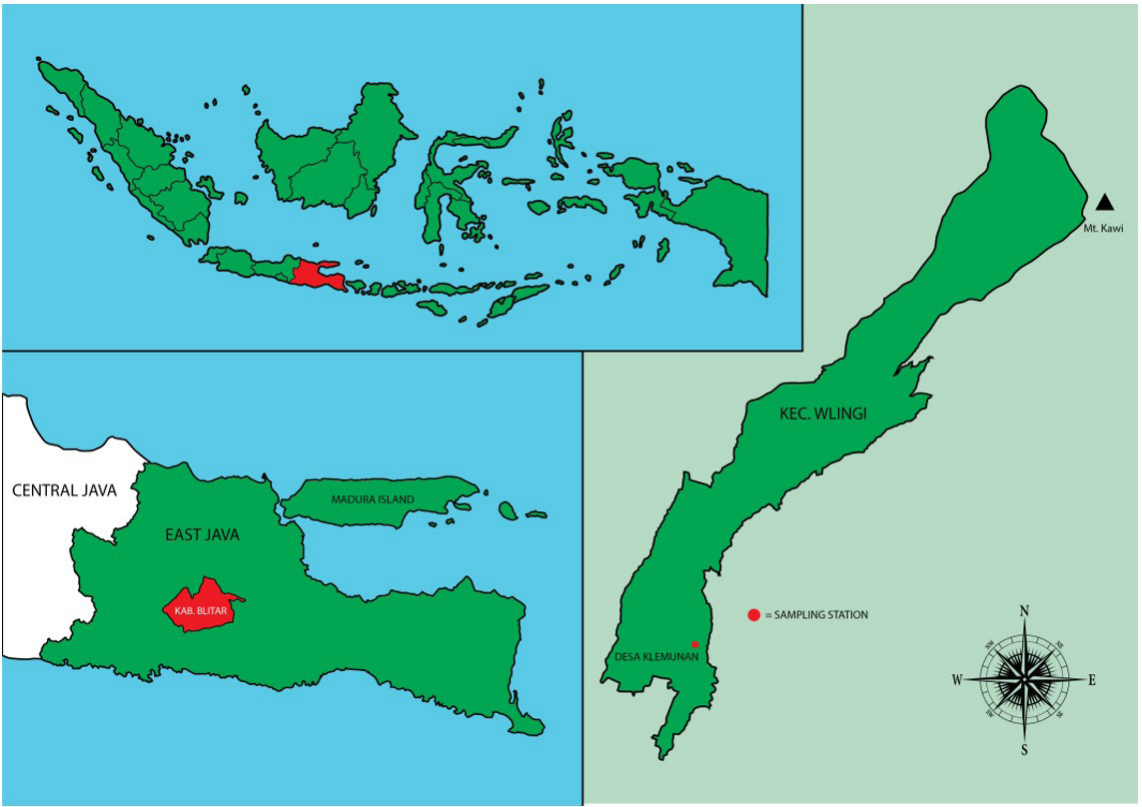
Geographical location of the sampling station.

Koi carp samples with a size of 5-10 cm were collected in June 2021 from Blitar, East Java, Indonesia (112°14'-112°28' East Longitude and 8°2'-8°8' South Latitude) (Figure 1).

### Fish and blood collection

The fish samples (n=60) were collected and transported by using a styrofoam box filled with ice from the field to the Laboratory of the Livestock and Fisheries Service of Blitar Regency as described in a previous study for ectoparasite examination (Morey et al., 2022). Before being packed in styrofoam, koi carp were anesthetized using clove oil and directly measured for blood glucose levels using an EasyTouch^™^, then packed per fish and labeled. Blood was drawn using a 1 mL syringe filled with 2% EDTA (Ethylene Diamine Tetra Acid) solution which was used as an anticoagulant. Koi carp blood sampling was done through the caudal vein. The caudal vein is under the vertebrae. A minimum of 0.1 ml of blood is taken and then inserted at the end of the test script that has been inserted into the digital blood glucose test kit. The results of blood glucose levels will appear on the screen of a digital blood glucose device in the form of numbers with units of mg/dL. The fish samples used in this study have found animal welfare based on Guidelines for the Use of Fishes in Research (AFS, 2014) and approved by Fisheries and Marine Faculty of Universitas Airlangga according to protocol number 1231, 2021.

### Examination of study parameter

The main parameters observed were blood glucose levels, ectoparasite prevalence, infestation, intensity, and water quality. Examination of ectoparasites in koi carp (n=60) was carried out using the native method by scraping on the surface of the body and gills (Yusni & Rambe, 2019). The scrapping results were observed using a binocular microscope (OLYMPUS Cx21) with a magnification of 100x. The degree of infestation was determined as the severity of the damage caused by the parasite to the host. Formula of the degree of ectoparasite infestation determination according to (Williams & Bunkley-Williams, 1996) was shown in Table 1.

**Table 1:** Formula of degree of parasite infestation determination.

Level of infestation (parasites)	Categories
<1	Very light
1-5	Light
6-10	Moderate
11-50	Medium
51-100	Heavy
101-999	Superheavy
>1000	Superinfection

The prevalence and intensity of ectoparasites were observed according to Bush et al. (1997), while blood measurement was conducted according to previous research methods (Eames et al., 2010). Prevalence is the total number of cases of a disease occurring at a certain time (Ayanful-Torgby et al., 2018). The prevalence was calculated using the following formula (Pawar, 2022):


$$\text{Prevalence =}\frac{\text{Number of infected fish}}{\text{Number of fish samples}}\text{x 100\%}$$ (1)

The ectoparasite intensity was calculated to determine the number of parasites in individuals or populations which was indicated by the average parasite value per host (Shaw et al., 2018). Intensity was calculated using the following formula (Hakim et al., 2019):


$$\text{Intensity =}\frac{\text{Number of parasites found}}{\text{Number of fish infested with parasites}}$$ (2)

The results of the examination of blood glucose levels with units of mg/dL and the degree of ectoparasite infestation are presented in the form of tables and figures to provide a descriptive picture.

### Water quality

Water quality measurement data follows the procedures from previous studies (Wiyoto & Effendi, 2020). The results of the water quality measurement in the koi carp pond soil obtained a temperature of 28^o^C, dissolved oxygen 4.71 mg/L, pH 8.6, and ammonia 0.05 mg/L.

### Statistical analysis

This study used an ANOVA test at 5% significance to determine differences in blood glucose values ​​of koi carp in each category of the degree of ectoparasite infestation to evaluate the rapid method. Prior to the ANOVA test, the normality test was carried out by Kolmogorov Smirnov and the homogeneity test was carried out by Levene's Test.

## Results

### Fish parasite examination

**Figure 2:**
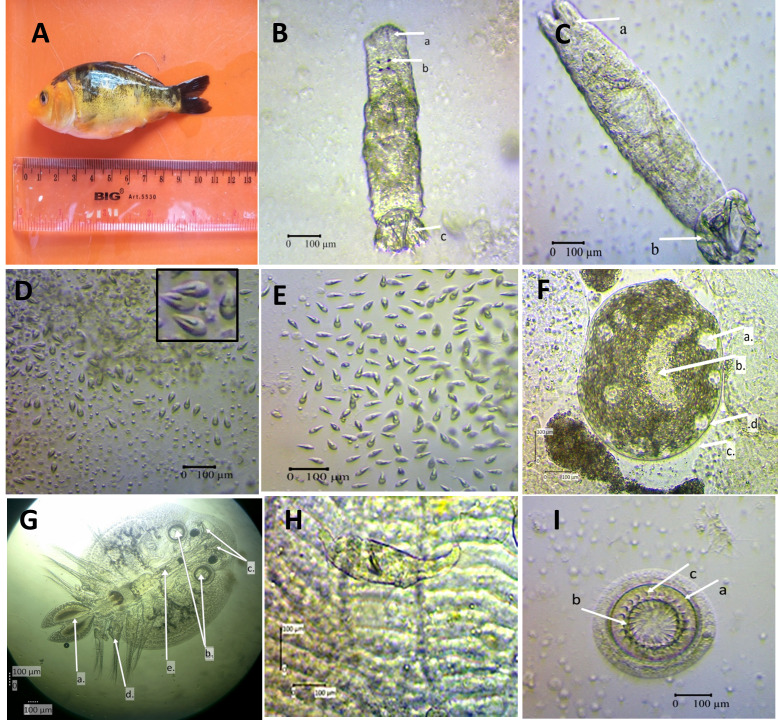
A. *Cyprinus carpio*; B. *Dactylogyrus* sp. (a. Opisthaptor; b. Eye spot; c. Hook), 100x; C. *Gyrodactylus* sp. (a. Opisthaptor; b. Haptor*)*, 100*x*; D. *Myxobolus* sp., 100x; E. *Thelohanellus* sp., 100x; F. *Ichthyophthirius* (a. Micronucleus; b. Macronucleus; c. Cilia; d. Membrant), 100x; G. *Argulus* sp. (a. Testes; b. Maxilla; c. Stylet; d. Leg; e. Proboscis), 40x; H. Skin infested by *Gyrodactylus* sp.; I. *Trichodina* sp. (a. Adhesive disc; b. Denticle ray; c. Denticle), 100x.

The results of the examination of ectoparasites found are *Trichodina, Dactylogyrus, Gyrodactylus, Myxobolus, Thelohanellus, Ichthyophthirius,* and *Argulus* (Figure 2). *Trichodina* showed the highest prevalence (100%) in this case while *Ichtyophthirius* was the lowest (13.3%) (Figure 2). Heavy levels of infestation showed in *Myxobolus* and *Thelohanellus.* In intensity level, *Thelohanellus* was the highest (93.8±16.3) while *Argulus* showed the lowest value (2.1±0.7) (Table 2).

**Table 2:** Fish parasite observation in this study.

Species Fish	Ectoparasite	Predilection	Prevalence (%)	Mean Intensity	Degree of infestation
*Cyprinus carpio (n=60)*	*Trichodina* sp.	Gills, skin	100	23.9±6.03	Medium
	*Dactylogyrus* sp.	Gills, skin	95	21.8±0.25	Medium
	*Gyrodactylus* sp.	Gills, skin	46.7	4.9±3.61	Light
	*Myxobolus* sp.	Gills, skin	31.7	75±25.03	Heavy
	*Thelohanellus* sp.	Gills, skin	13.3	93.8±16.3	Heavy
	*Ichthyophthirius*	Gills	6.7	2.3±2.5	Light
	*Argulus* sp.	Skin	15	2.1±0.7	Light

### Koi carp blood glucose profile

Measurement of blood glucose levels showed a different value in fish infested with parasites. The result showed that koi carp with light, medium, and heavy infestation of ectoparasites had blood glucose levels that were not significantly different (*p* = 0.715 > 0.05) (Table 3).

**Table 3:** Results of blood glucose level measurement based on the degree of parasite infestation. There was no significant difference in blood glucose for the three degrees of ectoparasitic infestation (*p* = 0.715 > 0.05).

Fish Samples	Degree of infestation	Blood glucose level		
		Mean ± SE (mg/dL)	F-value	*p*-value
*Cyprinus carpio* (n=60)	Light	99.242±6.592	0.337	NS
	Medium	86.333±26.168		NS
	Heavy	106.791±10.819		NS

SE: standard error; NS: not significant.

## Discussion

### Ectoparasite investigation and blood glucose response

The parasites found in this study were indeed ectoparasites in koi carp. *Trichodina, Dactylogyrus, Gyrodactylus, Myxobolus,* and *Thelohanellus* were infested in the gills and skin of koi carp (Elisafitri et al., 2021; Yanuhar et al., 2019; Zhang et al., 2022). Another study stated that *Ichthyophthirius* attaches to gills while *Argulus* is found in koi carp skin (Firdausi et al., 2020; Koyuncu, 2020). Gills are a favorite part for parasites to attach to. This is because the parasite uses the host molecule on the gills as a receptor and tries to avoid the host’s immune system (Scheifler et al., 2022). The presence of parasites in fish gills reduces the gill surface area, causes disturbances of hydromineral balance, increases ATP-ase expression and induces apoptosis in mitochondria-rich cells (Oğuz & Oğuz, 2020). This mechanism causes reduced oxygen entering the fish, the color of the gills becomes pale, uncertain movement, fade color, stress, and even mortality (Suliman et al., 2021). Besides gills, skin is a favorite place for parasites to attach. This is because the parasite attaches to the skin and gills of the host to get nutrients in the form of epidermal cells or even blood to develop (Faruk, 2018). The skin-infested parasite is the most abundant, diverse, and pathogenic group of parasites in both freshwater and seawater fish (Zhang et al., 2017).

### Infestation level of koi carp

This study also showed that the infestation of light, medium, and heavy were found in koi carp from Blitar. Previously, extreme parasite cases were reported in the Koi carp from Blitar which caused mortality of up to 90% (Maftuch et al., 2018; Soelistyoadi et al., 2020; Yanuhar et al., 2021). The presence of ectoparasite infestations on the body surface of fish could change the normal physiological function of host cells in infested organs and symptoms including lethargy, gill or skin lesions and increased mucus production (Jiang et al., 2017). If two or more parasites infest the same host or organ, there will be competition with each other for nutrients from the host which can affect the immunity and physiology of the host's body (Poulin, 2013). Fish will experience stress and overcoming this requires more energy sources than glucose. Fish divert energy normally used for growth to other physiological processes to maintain homeostasis (McNamara et al., 2013).

### The cause of parasitism

The phenomenon of parasitic attack in Blitar was probably caused by environmental factors such as water quality. The results of the water quality measurement in the koi carp pond were temperature 28^o^C, dissolved oxygen 4.71 mg/L, pH 8.6, and ammonia 0.05 mg/L. According to a previous study, the optimal water quality parameters for the survival of fish were having an oxygen content of more than 5 mg/L, pH of 6.5-8.5, and ammonia content of less than 0.05 mg/L (Noga, 2010). It was indicated that the water quality in the koi carp pond was not optimal for the growth of koi carp. Low dissolved oxygen and high temperature could be the main factors that caused the high infestation parasite in the soil pond where the koi carp are cultivated. In water conditions with warm temperatures (25-32^o^C), acidic pH, and low dissolved oxygen will encourage the proliferation of the parasite group (Saha et al., 2013). The poor water quality in Blitar for koi cultivation may be caused by the origin of the water source. Another study stated that koi cultivation in Blitar used springs, rainwater, river and irrigation water (Kilawati et al., 2020; Kartikasari et al., 2021). This water source had been polluted due to the activity of factory, household and livestock waste so its quality decreased (Sabila et al., 2022; Khopsoh et al., 2021; Hertika et al., 2021; Izzati & Retnaningdyah, 2022).

### Blood glucose profile as fish health indicator

This study showed the infestation of ectoparasites with light, medium, and heavy categories increases the blood glucose levels of koi carp. It was indicated that blood glucose profile could be used as a rapid method to determine the fish’s health. This finding is also the same as a previous study that found no change in haemotological parameters in fish infected with parasites (Fallah et al., 2015). Glucose is a monosaccharide of the aldohexose group and a necessary source of energy and carbon for most vertebrates including fish. Blood glucose concentration is widely used as key physiological indicator expressing the general health condition of fish (Endo & Wu, 2019). Glucose is the primary carbohydrate energy source of vertebrates and is stored as glycogen, an a-linked polymer, predominantly in the liver and muscles (‘animal starch’) (Shendurse & Khedkar, 2016). The average blood glucose level in this study indicated the value exceeds the limit. According to previous studies, the normal blood glucose level in fish is 40-90 mg/dL (Patriche, 2009). Several studies have shown the role of glucose as an indicator of the state of stress in fish due to ectoparasites such as *Trichodina*, *Cryptocaryon*, *Lernaea cyprinacea*, *Argulus foliaceus*, *Ceratothoa oestroides* (Mahasri et al., 2020; Fallah et al., 2015; Özdemir et al., 2016). In spite of the extended use of glucose as a common stress indicator, undefined and uncontrolled variables which may alter the response in the secretion of glucose must be considered, such as nutritional status, life stage, time since the last feeding, maturation or effects of swimming. Most of those factors are not directly considered stressors but have an effect on the glucose level which makes them a source of error (Martínez-Porchas et al., 2009; Malik et al., 2020).

## Conclusion

The result of this study provides information regarding the use of glucose as an indicator of fish health status regarding fish ectoparasite infestation. Changes in blood glucose values ​​can be used as an early indicator of impaired fish health due to parasites. This study also suggests that other variables such as nutritional status, life stage or feeding must be conducted to ensure the glucose role in parasite identification as a rapid method.

## References

[B001] Abidin Z, Setiawan B, Soemarno, Primyastanto M, Sulong A (2019). Ecological and socio-economic sustainability of ornamental fish business in minapolitan area of Blitar Regency, East Java, Indonesia. IOP Conf Ser Earth Environ Sci.

[B002] AFS (2014). Guidelines for the use of fishes in research.

[B003] Ayanful-Torgby R, Quashie NB, Boampong JN, Williamson KC, Amoah LE (2018). Seasonal variations in *Plasmodium falciparum* parasite prevalence assessed by varying diagnostic tests in asymptomatic children in Southern Ghana. PLoS One.

[B004] Azmi H, Indriyanti DR, Kariada N (2013). Identifikasi ektoparasit pada ikan koi (*Cyprinus carpio* L) di pasar ikan hias Jurnatan Semarang. Life Sci.

[B005] Bush AO, Lafferty KD, Lotz JM, Shostak AW (1997). Parasitology meets ecology on its own terms: Margolis et al. revisited. J Parasitol.

[B006] Domasevich MA, Hasegawa H, Yamazaki T (2022). Quality evaluation of Kohaku Koi (*Cyprinus rubrofuscus*) using image analysis. Fishes.

[B007] Eames SC, Philipson LH, Prince VE, Kinkel MD (2010). Blood sugar measurement in Zebrafish reveals dynamics of glucose homeostatis. Zebrafish.

[B008] Elisafitri M, Satyantini WH, Arief M, Sulmartiwi L (2021). Parasitic disease in Koi fish (*Cyprinus carpio*) in freshwater ponds with different densities in Sukabumi, West Java. IOP Conf Ser Earth Environ Sci.

[B009] Endo H, Wu H (2019). Biosensors for the assessment of fish health: a review. Fish Sci.

[B010] Fallah FJ, Khara H, Rohi JD, Sayadborani M (2015). Hematological parameters associated with parasitism in pike, *Esox lucius* caught from Anzali wetland. J Parasit Dis.

[B011] Faruk MAR (2018). Fish parasite: infectious diseases associated with fish parasite.

[B012] Firdausi AP, Rahman R, Mahadhika R, Sumadikarta A (2020). The ectoparasitic protozoa of koi fish (*Cyprinus carpio*) in Sukabumi. J Akuakultur Rawa Indones.

[B013] Hakim LN, Irawan H, Wulandari R (2019). Identification, intensity and prevalence of endoparasites in Silver Pompano *Tachinotus Bloch* at Plantation City Tanjungpinang. Intek Akuakultur.

[B014] Hertika AMS, Supriatna S, Darmawan A, Nugroho BA, Handoko AD, Qurniawatri AY (2021). The hematological profile of *Barbonymus altus* to evaluate water quality in the Badher bank conservation area, Blitar, East Java, Indonesia. Biodiversitas J.

[B015] Izzati FN, Retnaningdyah C (2022). Evaluation of river water quality based on biotic index of benthic macroinvertebrate as bioindicator (case study in Genjong River Wlingi Blitar East Java, Indonesia). Biotropika J Trop Biol.

[B016] Jiang D, Wu Y, Huang D, Ren X, Wang Y (2017). Effect of blood glucose level on acute stress response of grass carp *Ctenopharyngodon idella.*. Fish Physiol Biochem.

[B017] Kartikasari DPP, Bhawiyuga A, Kilawati Y, Maimunah Y, Muttaqin A (2021). Memanfaatkan internet of aquaculture dalam meningkatkan kualitas produksi pada kelompok pembudidaya ikan koi di Blitar. J Innov Appl Technol.

[B018] Khopsoh B, Faradila R, Lidiyawati A, Haryuni N, Lestariningsih L, Afrilia T (2021). Identifikasi bakteri *Escherichia coli* dari air minum unggas di Peternakan Layer. Musamus J Sci.

[B019] Kilawati Y, Maimunah Y, Muttaqin A, Kartikasari DP, Bhawiyuga A, Amrillah A (2020). Implementasi internet of aquaculture (IoA) untuk deteksi kualitas lingkungan secara cepat dalam upaya pemberdayaan kelompok pembudidaya ikan koi di Blitar. J Innov Appl Technol.

[B020] Kismiyati K, Subekti S, Inaya AFN (2015). The influence of papaya seed (*Carica papaya*) toward the damage eggs of *Argulus japonicus.*. J Ilm Perikan Kelaut.

[B021] Koyuncu C (2020). Koi balığı (*Cyprinus carpio*, Linnaeus, 1758) yetiştiriciliğinde *Argulus japonicus* (Thiele, 1900) enfestasyonu. Acta Aquat Turc.

[B022] Maftuch M, Sanoesi E, Farichin I, Saputra BA, Ramdhani L, Hidayati S (2018). Histopathology of gill, muscle, intestine, kidney, and liver on *Myxobolus* sp. infected Koi carp (*Cyprinus carpio*). J Parasit Dis.

[B023] Mahasri G, Widyastuti P, Sulmartiwi L (2011). Leucocyte profil of Koi fish (*Cyprinus carpio*) which infested by *Ichthyophthirius multifiliis* on the different infestation degree with cohabitation methode. J Ilm Perikan Kelaut.

[B024] Mahasri G, Yodharta IND, Novalisa D, Mukti AT (2020). Correlation between glucose level and protozoan ectoparasite infestation level of Humpback Grouper (*Cromileptes altivelis*) nursery in UPBL Situbondo, East Java. IOP Conf Ser Earth Environ Sci.

[B025] Makaras T, Razumienė J, Gurevičienė V, Šakinytė I, Stankevičiūtė M, Kazlauskienė N (2020). A new approach of stress evaluation in fish using β-d-Glucose measurement in fish holding-water. Ecol Indic.

[B026] Malik MA, Komarewar S, Dar SA (2020). Why fish is a natural diabetic animal?. World J Aquac Res Develop.

[B027] Martínez-Porchas M, Martínez-Córdova LR, Ramos-Enriquez R (2009). Cortisol and glucose: reliable indicators of fish stress?. Pan-Am J Aquat Sci.

[B028] McNamara KB, Van Lieshout E, Jones TM, Simmons LW (2013). Age‐dependent trade‐offs between immunity and male, but not female, reproduction. J Anim Ecol.

[B029] Morey GAM, Rojas CAT, Marin GAR, Guardia CTC (2022). Occurrence of *Eustrongylides* sp. (Nematoda: Dioctophymatidae) in fish species collected in the Peruvian Amazonia and its implications for public health. Acta Parasitol.

[B030] Noga EJ (2010). Fish disease: diagnosis and treatment.

[B031] Oğuz AR, Oğuz EK (2020). Histopathology and immunohistochemistry of gills of Van fish (*Alburnus tarichi* Güldenstädt, 1814) infected with myxosporean parasites. J Histotechnol.

[B032] Özdemir G, Çelik EŞ, Yilmaz S, Gürkan M, Kaya H (2016). Histopathology and blood parameters of Bogue Fish (*Boops boops*, Linnaeus 1758) parasitized by *Ceratothoa oestroides* (Isopoda: cymothoidae). Turk J Fish Aquat Sci.

[B033] Patriche T (2009). The importance of glucose determination in the blood of the cyprinids. Lucr Stiint Zooteh Biotehnol.

[B034] Pawar RT (2022). Studies on the prevalence and seasonal variation of *Gangesia* (*Gangesia*) *ramkai* (Pawar, 2008) from freshwater fish, *Wallago attu* (Bleeker). Int J Fauna Biol Stud.

[B035] Poulin R (2013). Explaining variability in parasite aggregation levels among host samples. Parasitology.

[B036] Sabila AT, Highall AR, Purbasari A, Perwiro D, Sulistywati E, Widianto AA (2022). Analisis pengaruh limbah Pabrik Gula Rejoso Manis Indo terhadap pencemaran lingkungan masyarakat Rejoso dan Umbuldamar. JIHI3S.

[B037] Saha H, Saha RK, Kamilya D, Kumar P (2013). Low pH, dissolved oxygen and high temperature induces *Thelohanellus rohita* (myxozoan) infestation in tropical fish, *Labeo rohita* (Hamilton). J Parasit Dis.

[B038] Scheifler M, Magnanou E, Sanchez-Brosseau S, Desdevises Y (2022). Host specificity of monogenean ectoparasites on fish skin and gills assessed by a metabarcoding approach. Int J Parasitol.

[B039] Shaw AK, Sherman J, Barker FK, Zuk M (2018). Metrics matter: the effect of parasite richness, intensity and prevalence on the evolution of host migration. Proc Biol Sci.

[B040] Shendurse AM, Khedkar CD, Caballero B, Finglas PM, Toldrá F (2016). Encyclopedia of food and health.

[B041] Soelistyoadi RN, Nurekawati AD, Setyawati D (2020). Morphology and sequencing of *Myxobolus koi* DNA that infects Koi fish (*Cyprinus carpio*) in Blitar Regency. J Aquac Sci.

[B042] Suarez-Bregua P, Guerreiro PM, Rotllant J (2018). Stress, glucocorticoids and bone: a review from mammals and fish. Front Endocrinol.

[B043] Suliman EAM, Osman HA, Al-Deghayem WAA (2021). Histopathological changes induced by ectoparasites on gills and skin of *Oreochromis niloticus* (Burchell 1822) in fish ponds. J Appl Biol Biotechnol.

[B044] Sulmartiwi L, Harweni S, Mukti AT, Triastuti J (2013). Influence use of bandotan (*Ageratum conyzoides*) to rate Koi fish (*Cyprinus carpio*) blood glucose after transportation. J Ilm Perikan Kelaut.

[B045] Williams EH, Bunkley-Williams L (1996). Parasites of offshore big game fishes of Puerto Rico and the western Atlantic.

[B046] Wiyoto W, Effendi I (2020). Analysis of water quality for mariculture in Moro, Karimun, Riau Islands with principal component analysis. J Aquac Fish Health.

[B047] Yanuhar U, Hardiono SA, Junirahma NS, Caesar NR (2021). Profile of *Myxobolus* infection in koi fish (*Cyprinus carpio*) gill tissue from Land Pond, Nglegok, Blitar Regency. IOP Conf Ser Earth Environ Sci.

[B048] Yanuhar U, Junirahma NS, Susilowati K, Caesar NR, Musa M (2019). Effects of probiotic treatment on histopathology of koi carp (*Cyprinus carpio*) infected by *Myxobolus* sp. J Phys Conf Ser.

[B049] Yusni E, Rambe E (2019). Identification of ectoparasites in fry Tilapia (*Oreochromis niloticus*) in aquaculture pond. IOP Conf Ser Earth Environ Sci.

[B050] Zhang B, Zhai Y, Liu Y, Gu Z (2017). *Myxobolus pseudowulii* sp. n. (Myxozoa: Myxosporea), a new skin parasites of yellow catfish *Tachysurus fulvidraco* (Richardson) and redescription of *Myxobolus voremkhai* (Akhmerov, 1960). Folia Parasitol (Praha).

[B051] Zhang XY, Yao X, Zhou F, Yang CZ, Liu Y (2022). Identification of *Thelohanellus pseudonikolskii* n. sp. and *Myxobolus koi* Kudo, 1920 from goldfish *Carassius auratus.*. Aquacult Rep.

